# Demyelinating Neurobrucellosis Presenting With Neuropsychiatric Manifestations

**DOI:** 10.1155/carm/2210460

**Published:** 2026-02-23

**Authors:** Mojtaba Varshochi, Fatemeh Ravanbakhsh Ghavghani

**Affiliations:** ^1^ Infectious and Tropical Diseases Research Center, Tabriz University of Medical Sciences, Tabriz, Iran, tbzmed.ac.ir; ^2^ Department of Infectious Diseases, Faculty of Medicine, Tabriz University of Medical Sciences, Tabriz, Iran, tbzmed.ac.ir

**Keywords:** CNS infection, neurobrucellosis, zoonosis

## Abstract

Neurobrucellosis with psychiatric manifestations is an uncommon manifestation of brucellosis. Herein, we report a 31‐year‐old male complaining of dizziness, nausea, malaise, imbalance, amnesia, psychosis, delusion, and impulsive and disorganized behavior. He was not responsive to antipsychotic treatment, and his brain MRI showed demyelinating hyperintensity foci in the periventricular and paraventricular areas and the subcortical areas of the right temporal and frontal lobes. His serum and cerebrospinal fluid serology were in favor of brucellosis, and he dramatically responded to anti‐*Brucella* medications. Although demyelinating neurobrucellosis with psychiatric manifestations is rare, it should be suspected in unresponsive patients coming from brucellosis‐endemic areas.

## 1. Introduction

Brucellosis, also known as undulant fever or Mediterranean fever, is a zoonotic infection that is caused by the genus *Brucella*. While humans are dead‐end hosts and humans rarely infect other humans, the major animal reservoirs of these nonmotile, aerobic, intracellular, and Gram‐negative bacteria are cattle, sheep, goats, and pigs [1]. With more than 500,000 new cases each year, brucellosis is the most common bacterial zoonosis worldwide. Contact with infected animals or the ingestion of unpasteurized and uncooked dairy and meat products are the main routes of transmission to humans; it is reported that as few as 10 to 100 *Brucella* can lead to brucellosis in humans [2]. Human brucellosis appears in acute (less than 8 weeks), subacute (less than 8–52 weeks), and chronic (above 52 weeks) phases [3]. The clinical manifestations of the acute phase of human brucellosis are similar to severe flu‐like symptoms [4]. In the subacute phase, the clinical manifestation of the disease is protean and less severe than in the acute phase; the subacute phase is due to incomplete or inappropriate antibiotic administration [3]. The prominent clinical presentation of chronic brucellosis is generalized musculoskeletal pain, emotional lability, nervousness, arthralgia, fatigue, and malaise [5].

Besides syphilis and tuberculosis, brucellosis is considered a “great imitator” due to the involvement of various organs [6]. The involvement of the skeletal system, cardiovascular system, gastrointestinal system, hematopoietic system, genitourinary system, and respiratory system, along with ocular and skin lesions, is reported following brucellosis [3, 7]. Neurobrucellosis constitutes a remarkable clinical challenge given its various clinical manifestations; neurobrucellosis can affect both peripheral and central nervous systems [8]. Meningitis, confusion, hypoesthesia, convulsions, hemiparesis, paraplegia, dysarthria, diplopia, and papilledema are the reported findings in patients with neurobrucellosis [9]. Neurobrucellosis can also present with psychiatric signs and symptoms. Behavioral changes, cognitive impairment, depression, and anxiety are the reported psychotic manifestations of neurobrucellosis [10].

Herein, we report a 31‐year‐old male from a brucellosis‐endemic area who presented with neuropsychiatric symptoms of chronic brucellosis. The current case report and literature review aim to shed light on the significance of neuropsychiatric aspects of chronic brucellosis, especially in patients coming from brucellosis‐endemic areas.

## 2. Case Presentation

**FIGURE 1:**
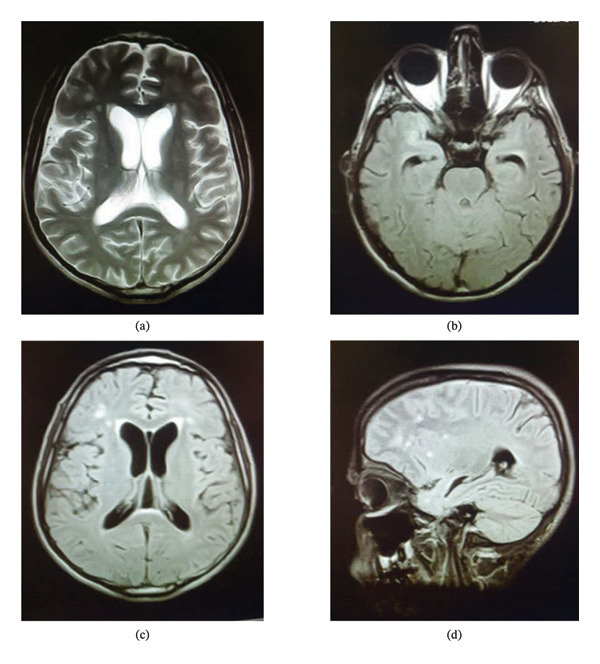
The brain MRI of the patient. (a) Hydrocephalus; axial T2W (TR/TE 5000/96) image without contrast, (b) hyperintensity lesion in the right temporal lobe; axial T1W (TR/TE 550/8.7) image without contrast, (c) demyelinating lesions in the right and left frontal lobes; axial T1W (TR/TE 9000/92) image without contrast, and (d) demyelinating lesions in the frontal lobe; sagittal T1W (TR/TE 9000/89) image without contrast.

A 31‐year‐old male rancher with no apparent past medical history had been complaining of nausea, vomiting, and generalized malaise since two years ago. He had experienced gradual weight loss over the past 2 years. Six months ago, his family members reported impulsive and disorganized behavior along with psychosis. Therefore, he was admitted to a psychiatric hospital with the initial diagnosis of schizophrenia and treated with olanzapine and metoclopramide. Following the initiation of the antipsychotic medication, his symptoms were exacerbated, and his family requested discharge by personal consent from the psychiatric hospital. Following the addition of imbalance and amnesia, a neurologist ordered an EEG and brain MRI. The EEG conclusion was generalized slow‐wave discharge. The brain MRI showed focal subcortical hyperintensity in the anterior aspect of the right temporal lobe with a similar, smaller hyperintensity at the superolateral aspect of the right frontal lobe. Also, hyperintense foci were found in the periventricular and paraventricular areas and subcortical white matter. In addition, mild communicating hydrocephalus was noted. These foci were demyelinating in nature, and there were no diffusion restrictions. The cervical MRI report did not demonstrate any pathological findings. The radiologist’s impression was a demyelination with a possible diagnosis of vasculitis (Figure 1).

Following impaired consciousness and pyrexia, the patient was admitted to the infectious ward at Imam Reza Hospital, a tertiary teaching hospital affiliated with Tabriz University of Medical Sciences. Because of his clinical signs and symptoms, the initial impression was meningoencephalitis. His vital signs at the time of admission were as follows: blood pressure: 105/60 mmHg, pulse rate: 82 bpm, respiratory rate: 14 breaths per minute, oral temperature: 38°C, and oxygen saturation without supplemental oxygen: 96%. He was not alert and not oriented to time, place, or person. He was agitated, and delusion and disorganized behavior were apparent. The flight of ideas was evident during the medical examination. Regarding the general appearance, he was cachectic. The heel‐to‐chin test was abnormal, and his tandem gait was impaired; however, the extraocular muscle functions were normal, and no nystagmus was found. The initial laboratory workups, i.e., CBC, ABG, urine analysis, blood culture, renal function tests, thyroid function tests, liver function tests, CRP, magnesium, potassium, phosphate, and calcium serum levels, were in the physiological range. The values for ALT, AST, and ALP were 11 IU/L, 15 units/L, and 64 IU/L, respectively. The ESR value was 30 mm/hour and the serum level of sodium was as low as 126 (mEq/L), which was corrected in the following days according to the related protocols. The HIV antibody, HBs antigen, HCV antibody, P‐ANCA, C‐ANCA, ANA, and anti‐dsDNA were negative. Following the suspicion of sarcoidosis, the ACE level was ordered, and its level was normal. The initial chest X‐ray demonstrated no pathological findings, and the total abdominopelvic ultrasonography only found a calcified focus with a diameter of 5.5 mm in the liver, which was consistent with old granuloma. Furthermore, the lumbar puncture was performed in an aseptic condition to study the cerebrospinal fluid. Following lumbar puncture, acyclovir, vancomycin, and ceftriaxone were immediately initiated with suspicion of infection in the central nervous system. The report of cerebrospinal fluid analysis was as follows: WBC: 67 (PMN: 70% and lymphocyte: 30%), RBC: 0, protein: 2 mg/dL, glucose: 48 mg/100 mL, LDH: 98 mg/dL, and ADA: 14 U/L. The Wright, Coombs Wright, and 2‐mercaptoethanol on the cerebrospinal fluid were 1:2560, 1:2560, and 1:1280, respectively. The Wright, Coombs Wright, and 2‐mercaptoethanol assays on the serum were repeated, and their results were 1:160, 1:320, and 1:160, respectively. After confirmation of brucellosis, rifampin and doxycycline were added to his treatment, and acyclovir and vancomycin were discontinued. In addition, antiedematous therapy for the patient’s mild communicating hydrocephalus was not initiated following a consultation with a neurologist.

After administration and continuation of the treatment, his nausea, vomiting, fever, amnesia, imbalance, and behavioral changes improved over a week after his admission to the infectious ward. Also, his recent memory loss was improved during admission to the hospital. After 1 month of admission, all the signs and symptoms were normalized, and he was discharged with oral medications.

## 3. Discussion

Neurobrucellosis is a challenging infectious disease due to its nonspecific range of symptoms. Based on a meta‐analysis study, the prevalence of neurobrucellosis is 4%; neurobrucellosis can be presented in the form of meningitis, seizure, sciatica, motor deficits, confusion, and psychological disturbances [4]. Neurobrucellosis can be identified in each phase of brucellosis. Despite the low mortality rate of neurobrucellosis, it has been reported that neurobrucellosis patients can develop neurological sequelae [9, 11]. The psychiatric manifestations of brucellosis are psychosis, agitation, nightmares, depression, amnesia, personality disorder, and euphoria [10]. Here, we report a 31‐year‐old male who presented with neuropsychiatric symptoms, i.e., behavioral changes, dizziness, and amnesia. This case report and literature review underscores the importance of neurobrucellosis in the differential diagnosis of patients presenting with neuropsychiatric signs and symptoms coming from *Brucella*‐endemic areas.

The mechanism by which *Brucella* affects the central nervous system is not completely understood. It is generally believed that reticuloendothelial system‐mediated *Brucella* bacteremia is the route to the meninges. Neurobrucellosis is the result of direct and indirect damage to the central nervous system; the endotoxin release and the activation of innate immune responses after the recognition of *Brucella* antigens, like lipoprotein and *Brucella* nucleic acids; cause the secretion of proinflammatory cytokines [12]. In this regard, IL‐6, IL‐1β, TNF‐α, CCL2, and CXCL1 cause reactive microgliosis. Indeed, astrogliosis and reactive microgliosis are the signs of brucellosis‐associated central nervous system inflammation [13]. Brain imaging in neurobrucellosis can be normal or demonstrate an inflammatory process or a vascular insult. Al‐Sous et al. have shown that patients with neurobrucellosis can have normal brain MRI, basal meningeal enhancement, granuloma of the suprasellar region, and diffuse white matter changes [14]. Based on the study by Jiang et al., the brain MRI of neurobrucellosis can be divided into five types, i.e., meningitis, meningoencephalitis, demyelination, abscess, and pseudotumor [15]. In this regard, demyelinating presentation of neurobrucellosis is very rare [16]. Our case demonstrated demyelinating hyperintensive foci in the anterior aspect of the right temporal lobe, the superolateral aspect of the right frontal lobe, and periventricular and paraventricular parts. The involvement of the temporal lobe can be related to his reported memory disorder, and the involvement of the frontal lobe can be related to his behavioral changes.

The definitive diagnostic approach is the isolation of specific bacteria from blood, bone marrow, cerebrospinal fluid, or other tissue. Serological assays are performed when bacterial isolation is not possible. The tube agglutination test, which measures antibodies against smooth lipopolysaccharides, and the 2‐mercaptoethanol test, which tests for immunoglobulin G for assessing active infection, are the popular serological assays for human brucellosis diagnosis. The diagnosis of human brucellosis is based on the isolation of *Brucella* species from human samples or the combination of a compatible clinical picture with the identification of specific antibodies above the threshold and/or at least a 4‐fold increase in antibody titer in serum after 2–3 weeks [5]. Regarding neurobrucellosis, the yield of culture from the cerebrospinal fluid and blood is low [9]. Therefore, serological assays are the main diagnostic methods. Based on the study by Guven et al., neurobrucellosis diagnosis is based on the positive serum brucellosis serology with the presence of one of the following: (I) signs and symptoms in favor of neurobrucellosis (neck stiffness, confusion, depression, personality changes, severe headache, insomnia, amenorrhea, incontinence, and any other neurological manifestations), (II) isolation of bacteria from cerebrospinal fluid and/or serological positivity in the cerebrospinal fluid, (III) lymphocytosis, decreased glucose, and increased protein levels in the cerebrospinal fluid, (IV) findings in favor of brucellosis in brain MRI or CT scan [11]. Ay et al. have reported a case of neurobrucellosis presented with a stroke in a 28‐year‐old male patient [17]. In addition, neurobrucellosis can be presented with upper motor signs with bilateral sensorineural deafness [18]. Shah et al. have reported a case of neurobrucellosis with acute psychosis and hearing loss [19]. Khademi et al. have reported a case of neurobrucellosis with the manifestations of hyperacute headache and behavior changes [20]. Psychosis is a rare complication of brucellosis and has not yet been clarified. Previous reports have demonstrated that demyelinating neurobrucellosis is most commonly presented with neurological signs and symptoms, like ataxia, positive Romberg’s sign, dizziness, and diplopia [21, 22]; however, our patient presented with psychosis, delusion, and impulsive and disorganized behavior, along with dizziness, imbalance, and amnesia. There have been only a few previous reports of *Brucella*‐induced psychosis in the scientific literature.

One notable case by Ghaffarinejad et al. involved a 26‐year‐old man from southern Iran who was admitted to a psychiatric hospital due to acute psychosis. For the past 6 months, this patient had been under treatment for brucellosis, experiencing fever, headache, and ataxia. During the treatment, he developed psychotic symptoms that responded to antipsychotic treatment. MRI during his initial admission revealed unidentified bright areas in the basal ganglia on T2‐weighted images [23]. Similarly, our patient developed psychotic symptoms a few months after the onset of general symptoms of brucellosis; however, he did not respond to antipsychotic treatment alone, and his condition even worsened. Remarkably, after starting antibrucellosis therapy, he showed significant improvement in his psychotic symptoms and other clinical manifestations.

In another study conducted by Zhang et al., a 48‐year‐old man presented with headaches, bilateral hearing loss, and psychosis persisting for 6 months. His MRI showed abnormal signals in the right frontal cortex and subcortical white matter, along with slight thickening and enhancement adjacent to the meninges [24]. In our patient, the MRI findings were suggestive of demyelinating neurobrucellosis, as indicated by hyperintensity foci in the periventricular and paraventricular regions, as well as in the subcortical areas of the right temporal and right frontal lobes.

Regarding the laboratory findings, the serology of serum and cerebrospinal fluid was positive for brucellosis, and there were high protein levels in the cerebrospinal fluid. Of interest, the patient was responsive to anti‐*Brucella* medications, and his symptoms were dramatically improved during hospitalization. Collectively, because the features of periventricular and paraventricular lesions in this patient are not in line with Dawson’s fingers characteristics, the CSF analyses showed highly positive neurobrucellosis‐related serology, and the patient dramatically responded to antibrucellosis medications, we believe that demyelinating neurobrucellosis is the cause of the patient’s signs and symptoms rather than multiple sclerosis. Besides, all the signs and symptoms of the patients had resolved over 2 weeks, and after 2 years of telemedicine follow‐up, we confirmed the complete resolution. However, due to the long distance, we did not have access to the patient specimen to run CSF analyses for multiple sclerosis.

## Funding

No funding was received for this manuscript.

## Ethics Statement

The patient was informed and signed written consent before writing this de‐identified case report. This study was approved by the Ethics Committee of Tabriz University of Medical Sciences (IR.TBZMED.REC.1403.765).

## Conflicts of Interest

The authors declare no conflicts of interest.
